# Germinal Center vs Extrafollicular Responses in Systemic Autoimmunity: Who Turns the Blade on Self?

**DOI:** 10.1016/bs.ai.2024.02.002

**Published:** 2024-03-06

**Authors:** Yuke He, Carola G. Vinuesa

**Affiliations:** 1 China-Australia Centre for Personalised Immunology (CACPI), Department of Rheumatology, Renji Hospital, School of Medicine, Shanghai Jiao Tong University (SJTUSM), Shanghai, 200001, China; 2 Francis Crick Institute, 1 Midland Rd, London NW1 1AT, United Kingdom

## Abstract

Spontaneously formed germinal centers (GCs) have been reported in most mouse models of human autoimmune disease and autoimmune patients, and have long been considered a source of somatically-mutated and thus high affinity autoantibodies, but their role in autoimmunity is becoming increasingly controversial, particularly in the context of systemic autoimmune diseases like lupus. On the one hand, there is good evidence that some pathogenic lupus antibodies have acquired somatic mutations that increase affinity for self-antigens. On the other hand, recent studies that have genetically prevented GC formation, suggest that GCs are dispensable for systemic autoimmunity, pointing instead to pathogenic extrafollicular (EF) B-cell responses. Furthermore, several lines of evidence suggest germinal centers may in fact be somewhat protective in the context of autoimmunity. Here we review how some of the conflicting evidence arose, and current views on the role of GC in autoimmunity, outlining mechanisms by which GC may eliminate self-reactivity. We also discuss recent advances in understanding extrafollicular B cell subsets that participate in autoimmunity.

## 1 Introduction

Germinal centers (GCs) are specific microstructures that develop in secondary lymphoid organs under antigen stimulation. B cells within GCs undergo clonal expansion, somatic hypermutation (SHM), selection, and eventually differentiate to affinity-matured plasma cells (PCs) or memory B cells. GC B cells are important source of high-affinity antibody required by long-term protective immunity.

GC is morphologically divided into two compartments: a dark zone (DZ) filled with dividing B cells (first called centroblasts) and a light zone (LZ) enriched with centrocytes (non-dividing progeny of centroblasts) (MacLennan, 1994;Victora and Nussenzweig, 2022). Activated B cells that have bound antigen and received cognate help form follicular helper T (T_FH_) cells at the T: B borders (Johnston et al., 2009;Nurieva et al., 2009;Yu et al., 2009). T_FH_ cells are a specialized subset of CD4 T cells characterized by expression of transcription factor Bcl6 and surface markers CXC-chemokine receptor 5 (CXCR5), inducible costimulator (ICOS) and programmed cell death protein 1 (PD-1) (Johnston et al., 2009;Nurieva et al., 2009;Yu et al., 2009). Upon priming, activated B cells upregulate activation-induced cytidine deaminase (AID) that allows immunoglobulin isotype switching and commence cell division at the periphery of the follicle (Allen, Okada and Cyster, 2007;Roco et al., 2019). Those B cells that downregulate EBI-2 migrate towards the center of the follicle where they undergo rapid clonal expansion as B blasts before moving to the pole of the follicle closer to the T zone and differentiate into centroblasts, giving rise to the dark zone (Pereira et al., 2009). Proliferating centroblasts further upregulate the expression of, AID, which introduces mutations into the Ig variable region genes and thereby changes antibody affinity and specificity, a process termed somatic hypermutation (SHM) (Berek, Berger and Apel, 1991;Griffiths et al., 1984;Jacob et al., 1991).

**Figure 1:**
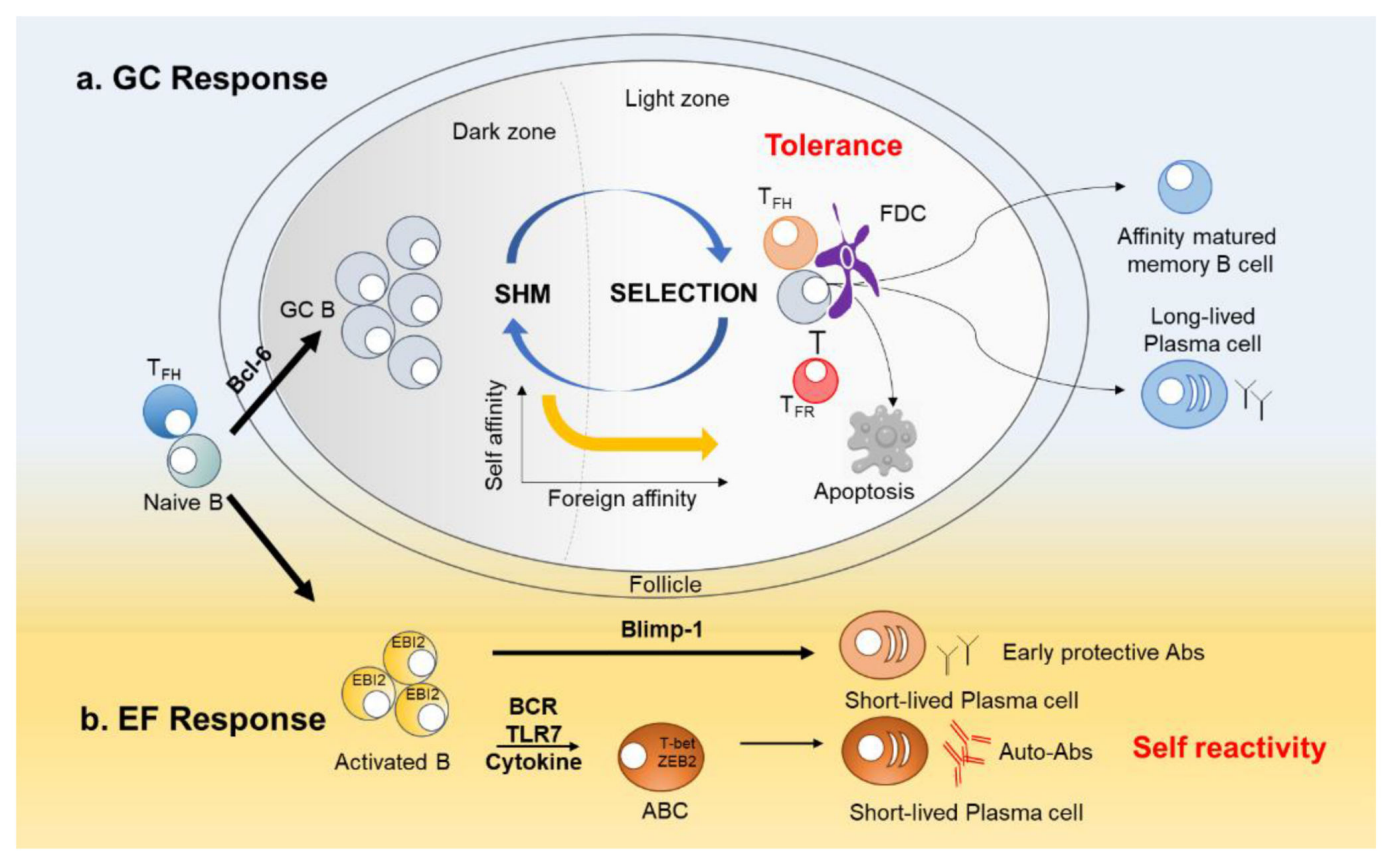
Germinal center and extrafollicular pathways in plasma cell differentiation. a. Germinal center (GC) response. GC response incorporates B cell clonal expansion, somatic hypermutation (SHM) and selection, and is the source of affinity matured memory B cells and plasma cells. GC selection is regulated by the balance of two T cell subsets: T follicular helper (T_FH_) cells that promote GC responses and T follicular regulatory (T_FR_) cells that inhibit the response. Self-reactive cells accidentally generated in the GC are safely confined in GC and are either cleared by apoptosis, or undergo new rounds of SHM and selection, thus eliminating self-affinity while increasing affinity towards foreign antigens if present. b. Extrafollicular (EF) response. Blimp-1 antagonizes the pro-GC signaling of Bcl-6 and promotes the development of short-lived PCs in EF sites (Shaffer et al., 2002). A subset of EF B cells known as age-associated B cells (ABCs or DN2 (IgD- CD27-) B cells are expanded in autoimmunity, the frequency of which correlates with autoantibodies and disease severity. The development of ABCs requires signals through the B cell receptor (BCR), TLR7, and cytokines such as IFN-γ and IL-21. ABCs differentiate readily into plasma cells and are considered a major source of autoantibodies.

Mutated dark zone B cells then exit cell cycle and move to LZ where they can bind antigen held in the form of immune complexes by a network of follicular dendritic cells (FDCs) that localizes in LZ (Kosco-Vilbois and Scheidegger, 1995). LZ B cells take up and process this antigen to present it to germinal center T_FH_ cells. GC T_FH_ cells govern B cell positive selection with GC and allow for optimal affinity-matured antibody responses. B cell clones with enhanced affinity tend to have an advantage in their competition for T_FH_ cell help and exit GCs, differentiating into memory B cells and long-lived PCs (Figure 1). A proportion of cells recycles to the dark zone to undergo further rounds of SHM. GC B cells can thus shuttle back and forth between the two zones through CXCR4/CXCR5-mediated migration (Allen et al., 2004).

This process is regulated by the balance of two T-cell subsets, T_FH_ cells that promote GC responses and T follicular regulatory (T_FR_) cells that limit selection and differentiation of self-reactive B cells and terminate GC reactions (Chung et al., 2011;Gonzalez-Figueroa et al., 2021;Jacobsen et al., 2021;Linterman et al., 2011).

GCs are not the sole environment in which B cells participate in humoral immunity. Upon encountering antigens, proliferating B cells that maintain EBI-2 expression migrate to extrafollicular (EF) regions and initiate antibody responses (Hannedouche et al., 2011;Liu et al., 2011;Pereira et al., 2009). Blimp-1 promotes the development of short-lived PCs in EF sites and can counteract pro-GC signaling by Bcl-6 (Shaffer et al., 2002). The EF pathway enables rapid differentiation of B cells to short-lived PCs, that provides fast protection against pathogens. Distinction between GC and EF contributions to B cell self-reactivity is essential for understanding of autoimmune pathogenesis and subsequent targeted therapies.

Over the last decade, many pieces have been added to the puzzle of autoantibody responses, although we still do not have a complete picture. New evidence from improved understanding of B cell responses and availability of mice that cannot form germinal centers suggests that GCs are dispensable for the initiation and maintenance of systemic autoimmunity. An extrafollicular B cell response, which lacks the required tolerance check-points, is likely to be the key source of autoimmunity. In this review, we outline the current models of GC-independent autoimmunity, and the factors that maintain GC self-tolerance. We also discuss the likelihood that GCs serve to constrain self-reactive B cells while defending against foreign antigens and review the extrafollicular B cell subsets that are emerging as prime suspects of pathogenic autoantibody production.

## 2 Assumptions that led to a pathogenic interpretation of spontaneous GCs

For the last two decades, the field of autoimmunity tacitly accepted a pathogenic role of germinal centers in autoantibody generation based on assumptions and interpretation of facts in the context of an incomplete understanding of the events that occur inside or outside of germinal centers. Here we outline some of the observations that led to the conclusions that autoantibodies arose in germinal centers.

A) Most lupus mouse models develop spontaneous GCs. Since a report in 2001 describing spontaneous GCs in young lupus-prone mice across a variety of genetic backgrounds (Luzina et al., 2001), multiple studies have reported similar findings across multiple models of human autoimmunity, contributing to the view that GCs promote autoimmunity. Most of these studies however have only provided indirect evidence of association between GCs and autoimmunity. However, studies have since been published and are described below in which either mice develop antinuclear antibodies in the absence of spontaneous germinal centers (Deng et al., 2017;Jang et al., 2019;O’Neill et al., 2011;Schwickert et al., 2019;Soni et al., 2020;Zhang et al., 2019), or lupus prone-mice that normally develop spontaneous GCs continue to produce autoantibodies despite genetic abrogation GC formation (Brown et al., 2022).
B) Causal relationship between T_FH_ overactivity and systemic autoimmunity. After the first association was made in *Roquin^san^* mice between increased T_FH_ cell numbers and lupus-like autoimmunity in 2005 (Vinuesa et al., 2005), followed by demonstration of a causal relationship (Linterman et al., 2009), a myriad of reports emerged collectively describing a near universal association between the magnitude of spontaneous GC reactions and baseline T_FH_ activation and manifestations of autoimmunity (Domeier, Schell and Rahman, 2017). Whilst it is likely that controlling T_FH_ cell numbers and reactivity via thymic selection is an important checkpoint against autoimmunity in GCs that may be breached in the presence of certain genetic susceptibility, it is also possible that the role of dysregulated T_FH_ cells in promoting autoimmunity does not occur within GC reactions. When T_FH_ cells were discovered to be an independent cell subset in 2009 (Johnston et al., 2009;Nurieva et al., 2009;Yu et al., 2009), their main function was considered to be their ability to drive and maintain GCs and this reinforced considering GCs pathogenic (Vinuesa, Sanz and Cook, 2009). It later became clear that T_FH_ cells played a near equal important function in driving extrafollicular responses (Lee et al., 2011), including formation of age-associated B cells (ABCs) (Song et al., 2022), thought to be pathogenic in lupus. Additional extrafollicular peripheral helper T cell subsets have also been identified and shown to play important roles in driving autoreactive B cell responses as described below (Marks and Rao, 2022;Odegard et al., 2008;Rao et al., 2017).
C) Pathogenic antibodies are nearly always isotype switched and isotype switching was until recently considered to occur predominantly in GCs. Except for a few exceptions including IgM antibodies in Guillain Barre Sd (Kaida et al., 2001), isotype switched antibodies are more pathogenic for their increased ability to fix complement and activate myeloid cells via their Fc gamma receptors (Takai, 2002). For decades, expression of AID – the enzyme required for both isotype switching and somatic hypermutation - had been considered to be a feature of GCs and this and other lines of evidence had led to the assumption that isotype switching mainly occurred in GCs. Indeed, it was not until 2019 that definitive evidence was provided demonstrating that isotype switching occurs prior to expression of Bcl-6 and the onset of somatic hypermutation (Roco et al., 2019), thus prior to B cells entering GCs. In terms of self/foreign discrimination, the GC pathway is likely to be more stringent and refined than the extrafollicular pathway, due to the requirement for T_FH_ cell help prior to differentiation into plasma cells. In the case of an extrafollicular response, a second signal delivered by a TLR ligand is sufficient as a second signal for a B cell to become a plasma cell in the absence of T cell help (Eckl-Dorna and Batista, 2009).
D) Multiple reports have described that pathogenic autoantibodies carry somatic mutations, and somatic hypermutation is generally associated with GC origin. These include early reports in the late 90s of somatic mutations that endow B cells with self-reactivity towards DNA (Ray, Putterman and Diamond, 1996), others directed against skin autoantigens (Di Zenzo et al., 2012), and more recent reports describing high affinity autoantibodies as a consequence of somatic mutations in 9G4 antibodies from SLE patients (Gomez-Banuelos et al., 2023;Ota et al., 2023). Given the stochastic nature of SHM, such self-reactive B-cell clones have long been assumed to be an inevitable by-product of GCs. However, somatic mutation, albeit limited, has been suggested to also occur extrafollicularly in the context of chronic bacterial infections such as *Salmonella* (Di Niro et al., 2015;Shlomchik et al., 1987) and in lupus-prone MRL.lpr mice in which self-reactive plasmablasts localize outside follicles (Sang et al., 2014;William et al., 2002). While these studies are compelling, definitive evidence of SHM requires genetic manipulation that prevents the development of GC responses in these models.

## 3 Evidence of GC-independent systemic autoimmunity

Certain models of autoimmune disease are accompanied by the presence of spontaneous GCs, whose precise role in autoimmunity remains unclear. In recent years, there has been increasing evidence that lupus may occur in a GC-independent manner in mice (Table 1) and humans.

### 3.1 Autoimmunity without spontaneous GC formation

#### cGVHD

The coexistence of autoantibodies and inadequate GC responses was firstly reported in chronic graft-versus-host disease (cGVHD) (Deng et al., 2017). After hematopoietic cell transplantation (HCT) using grafts containing a small number of C57BL/6 splenocytes, BALB/c recipients develop cGVHD, with elevated dsDNA-IgG and IgG deposition but less or no GC formation (Deng et al., 2017).

#### Ikaros deficiency

Ikaros, a transcription factor encoded by *Ikaros family zinc finger 1 (IKZF1)*, functions as a regulator of differentiation, proliferation and self-tolerance in lymphocytes. *IKZF1* is one of SLE risk genes identified by genome-wide association studies (GWAS) (Van Nieuwenhove et al., 2018), and conditional deficiency of *IKZF1* in B cells leads to systemic autoimmunity in mice (Schwickert et al., 2019). Deletion of Ikaros stringently blocks GC formation, and it is proposed that a Myeloid differentiation primary-response protein-88 (MyD88)-dependent extrafollicular response is responsible for the autoimmune phenotype (Schwickert et al., 2019).

#### Bach2 deficiency

BACH2 is a transcription repressor required for class switch recombination (CSR) and SHM (Muto et al., 2004). Mice lacking Bach2 develop lupus nephritis but fail to form GCs. *Bach*2-deficient B cells tend to localize outside the follicle and induce lupus via a T cell-dependent extrafollicular pathway (Jang et al., 2019).

#### Ship deficiency

SHIP is an inositol 5’ -phosphatase that acts as a negative regulator of immune responses. B cell-intrinsic Ship-deficiency (ShipΔB) causes a lupus-like disease in mice, which exhibit a compromised GC response, with self-reactivity thought to originate primarily from extrafollicular CD11c+Tbet+ ABCs (O’Neill et al., 2011;Zhang et al., 2019).

#### Dnase1l3 deficiency

Deoxyribonuclease 1 like 3 (DNASE1L3) is a secreted DNA endonuclease responsible for degrading antigenic DNA. Loss of function (Lof) mutations in *DNASE1L3* have been identified as causes of monogenic lupus (Al-Mayouf et al., 2011). *Dnase1l3-/-* mice do not show excessive expansion of GCs; instead, type I interferons secreted by plasmacytoid dendritic cells (pDCs) are thought to promote extrafollicular B cell differentiation and are required for the autoimmune phenotype (Soni et al., 2020). Combined deficiency of TLR7 and TLR9 has been shown to remove self-reactivity in this model (Soni et al., 2020).

### 3.2 Most lupus models require TLR7 signaling, but TLR7-dependent lupus does not require GC

Enhanced TLR7 signaling is a key mechanism in lupus pathogenesis and is becoming a hotspot for research and therapy. Increased TLR7 activation was reported in B cells from the majority of SLE patients (Jenks et al., 2018) and *TLR7* gain-of-function was subsequently identified as a cause of human monogenic form of lupus (Brown et al., 2022). More recently, variants in *UNC93B1* that disrupt degradative sorting of TLR7 (Mishra et al., 2024;Wolf et al., 2024) have also been shown to cause human lupus, underscoring the importance of this pathway in human SLE.

In mice, TLR7 overactivity appears to explain lupus disease in a large number of models. In some of these, lupus results from direct variation of TLR7 gene dose, or enhanced nucleic acid sensing function. These models include *BXSB Yaa* mice that harbour a chromosomal segment duplication from X onto Y that contains TLR7 (Pisitkun et al., 2006;Soni et al., 2014)*, Tlr7* transgenic (tg) mice (Walsh et al., 2012), TLR7-agonist (imiquimod) induced lupus (Hirobe et al., 2022;Voss et al., 2022), and *kika* mice that carry a *TLR7* gain-of-function variant found in a girl with SLE (Brown et al., 2022). In another group of lupus models in which *Tlr7* gene expression is intact, disease pathogenesis is also dependent on TLR7 signaling. Models in this group include 564 Igi tg mice (Berland et al., 2006), MRL.Faslpr (MRL/lpr) (Cosgrove et al., 2023;Shlomchik et al., 1987;William et al., 2002), Tnip1-deficient mice (Nanda et al., 2019), Unc93b1-deficient mice (Fukui et al., 2011), NZM2410-derived B6.Sle1.Sle2.Sle3 (TC) mice (Sang et al., 2014), and BAFF tg mice (Groom et al., 2007). Virtually all the mouse models in both groups show robust spontaneous GC formation, and deletion of TLR7 abrogates GC formation and autoimmune phenotypes (Christensen et al., 2006; Soni et al., 2014).

Toll-like receptor 7 (TLR7) signaling in B cells themselves has been shown to be required for spontaneous GC formation (Brown et al., 2022; Peterson et al., 2022; Soni et al., 2014) and fate-mapping of GC-derived B cells has revealed that TLR7-induced self-reactive B cells can be enriched in GCs (Castrillon et al., 2023;Degn et al., 2017). Besides inducing GCs, TLR7 overactivation is also associated with expansion of extrafollicular CXCR5-CD11c+Tbet+ B cells (known as IgD-CD27- DN2 B cells or age-associated B cells (ABCs)) as well as expansion of follicular and extrafollicular helper T cells (Brown et al., 2022; Jenks et al., 2018).

Whilst the evidence in mice described above has long suggested a pathogenic role of GCs in Tlr7-dependent lupus, definitive evidence on the role of such spontaneous GCs has only been obtained recently. By genetic manipulation of mice to prevent B cells from forming GCs (CD23^Cre^ *Bcl6*^flox^ mice), it has been found across three Tlr7-dependent lupus mouse models – *kika*, 564 Igi and imiquimod-induced lupus - that the absence of GC does not alleviate the autoimmune phenotype (Brown et al., 2022;Voss et al., 2022). Instead, defective GC formation leads instead to a more robust expansion of pathogenic ABCs and plasma cells and comparable or greater antinuclear antibody titres. These studies provide definitive evidence that TLR7-mediated autoimmunity can occur in a GC-independent manner and the extrafollicular response can be an important source of pathogenic B cells and autoantibodies.

## 4 GCs as a gatekeeper of B cell tolerance

The stochastic nature of SHM makes it possible to generate self-reactive B-cells, yet in healthy individuals, those cells can normally be effectively removed in GCs thanks to the existence of several tolerance checkpoints (Table 2).

### 4.1 B cell negative selection in germinal centers

#### BCR-mediated clonal deletion

It was discovered more than 20 years ago that GC B cells undergo waves of apoptosis after encountering soluble self-antigens (Pulendran et al., 1995;Shokat and Goodnow, 1995). The pro-survival protein Bcl-2 is involved in regulating the negative selection of GC B cells: overexpression of Bcl-2 significantly reduces apoptosis of GC B cells (Pulendran et al., 1995;Smith et al., 2000).

#### FAS-mediated cell apoptosis

GC B cells exhibit high levels of death receptor FAS, and deletion of FAS in B cells results in fatal lymphoproliferation (Hao et al., 2008;Smith, Nossal and Tarlinton, 1995). FAS is dispensable for soluble antigen-induced clonal deletion of GC B cells (Smith, Nossal and Tarlinton, 1995), and instead is pivotal for the control of “rogue” GC B cells that escape affinity selection and readily differentiate into PCs (Butt et al., 2015).

#### T_FR_-mediated inhibition of self-reactive B cell differentiation into plasma cells

T_FR_ cells are a population of Bcl6+Foxp3+ double-positive T cells that localize to the GC. They share some features with conventional T_FH_ and T_REG_ cells, but are distinct from both. T_FR_ cells may counteract the selection signal provided by T_FH_ cells, terminate the GC B response, and inhibit plasma cell differentiation (Chung et al., 2011;Jacobsen et al., 2021;Linterman et al., 2011;Merkenschlager et al., 2023;Wollenberg et al., 2011). Loss of T_FR_ cell control results in expansion of non-antigen (Ag)-specific B cells in GC (Linterman et al., 2011), as well as autoimmunity and allergy characterized by self-reactive IgE and IgG1 (Clement et al., 2019;Fu et al., 2018;Gonzalez-Figueroa et al., 2021;Xie et al., 2019). T_FR_ cells exert their tolerizing effects through their expression of cytotoxic T-lymphocyte-associated protein 4 (CTLA4) (Wing et al., 2014), PD-1 (Sage et al., 2013) and neuritin (Gonzalez-Figueroa et al., 2021). Neuritin can be taken up by B cells and inhibit their differentiation into PCs (Gonzalez-Figueroa et al., 2021), maintaining high BCL6 expression. Thus, TFR cells may prevent selection of self-reactive GC B cells into the periphery, maintain them in GC to undergo further rounds of mutation and T_FH_-mediated selection.

#### Macrophage clearance of apoptotic cells

GCs are sites of massive apoptosis. Tingible body macrophages (TBMs) are professional scavengers of apoptotic debris in GCs, preventing the activation of autoimmunity by self-nucleic acids decorating the apoptotic bodies. TBMs are derived from follicle-resident macrophage precursors and are triggered by apoptotic debris in situ (Grootveld et al., 2023). Milk fat globule epidermal growth factor (EGF) factor 8 (MFG-E8) is a soluble bridging protein that binds an apoptotic “eat me” signal phosphatidylserine, and promotes the phagocytosis of apoptotic debris by TBMs in GCs (Hanayama et al., 2004). Mice lacking the tyrosine kinases Tyro, Axel and Mer in macrophages cannot take up apoptotic GC B cells and develop lupus-like disease (Lu and Lemke, 2001).

### 4.2 Control of positive selection

Strict control of T_FH_ mediated B cell positive selection is important to prevent excessive autoimmunity (Pratama and Vinuesa, 2014). Quality control of T_FH_ cells starts in the thymus, where their precursors undergo negative selection resulting in elimination of T cells carrying self-reactive TCRs, preventing provision of help to B cells presenting self-peptides. Studies in human tonsils tracking the fate of autoreactive 9G4 B cells showed that in healthy individuals, despite some entering GCs, few matured into centroblasts, and they were not selected into the memory B cell pool (Cappione et al., 2005;Richardson et al., 2013). By contrast, autoreactive 9G4 B cells were found in the memory compartment of SLE patients, suggesting a breach in the control of T_FH_-mediated positive selection.

An example of a GC tolerance checkpoint that limits T_FH_ cell expansion is posttranscriptional regulation of T cell mRNAs by Roquin. Roquin is an RNA-binding protein belonging to the family of E3 ubiquitin ligases. Mice homozygous for a loss-of-function mutation of Roquin (Roquin^san/san^ (*sanroque*)) exhibit substantial expansion of T_FH_ cells and develop systemic autoimmunity. Roquin is known to bind to and degrade target mRNAs including *Icos*, inhibiting the number and function of T_FH_ cells (Vinuesa et al., 2005). This regulatory mechanism adds to the finely tuned dynamic regulation of T_FH_ cells within the GC, which is essential to maintain optimal high-affinity and avoid self-reactivity of GC responses (Merkenschlager et al., 2021;Shulman et al., 2013).

### 4.3 B cell confinement in germinal centers

Although there is no physical barrier surrounding GC, B cells are equipped with G protein-coupled receptors (GPCRs), such as P2RY8 (Gallman et al., 2021;Lu et al., 2019;Muppidi, Lu and Cyster, 2015) and S1PR2 (Green and Cyster, 2012), which confine the B cells to the GC region, thus allowing them to undergo cyclic rounds of SHM and selection. P2RY8 has been shown to be important to confine B cells to GCs (Muppidi, Lu and Cyster, 2015;Muppidi et al., 2014). Its ligand GGG is expressed in the periphery of the follicle (Lu et al., 2019). Recently, a P2RY8 L275F de novo variant was identified in a child with lupus nephritis (He et al., 2022). The L275F variant increases B cell migration and egress of GC B cells. P2RY8 was downregulated in some B cell subsets of lupus patients, and low expression of P2RY8 correlated with nephritis and extrafollicular ABCs (He et al., 2022). Notably, TLR7 ligands but not TLR9 ligand nor a large suite of cytokines were able to downregulate P2RY8. Therefore, P2RY8 is a gatekeeper that confines B cells in GC, and in doing this, may also limit pathogenic extrafollicular self-reactivity.

### 4.4 Autoreactivity redemption by SHM

Self-reactive B cells that are not immediately deleted by apoptosis and can present foreign antigen to T cells with cognate reactivity to such foreign antigen (e.g. in the case of immunization with self-foreign antigen conjugates), can undergo clonal redemption via rounds of SHM that eliminate autoreactivity whilst preserving reactivity to the foreign antigen. Single cell SHM analysis of SW_HEL_ GC B cells (whose BCR binds hen egg lysozyme with high affinity) in mice expressing HEL as a neo-self antigen, revealed a rapid selection of BCR mutations that lead to a loss of autoreactivity to self (HEL) while presumably increasing affinity to a related or conjugated foreign antigen (Reed et al., 2016;Sabouri et al., 2014;Young, Lau and Burnett, 2022) . Studies of human antibody mutations by deep sequencing have also provided evidence that the antibody response to vaccinia virus can evolve from rhesus D alloantigen (RhD) responses, and IGHV4-34^+^ B cells can also mutate away from autoreactivity in GCs although they often also lose foreign reactivity in the process and are thus “unredeemable” (Reed et al., 2016) . Thus, the self-reactive cells accidentally produced in GCs, instead of being an obstacle to immunity, can serve as a pre-existing army against foreign antigens.

It should also be noted that SHM-mediated autoreactivity redemption relies on the in situ or proximal expression of high levels of self-antigens in GC. GC-derived B cells retain autoreactive potential to those antigens that are rare in GC, or tissue-specific antigens in distal organs (Chan et al., 2012). That is, although GCs have a series of measures to eliminate B-cell self-tolerance and greatly prevent systemic autoimmunity, GC-derived B cells still harbor pathogenic potential for organ-specific autoantigens and may thus still cause organ-specific autoimmunity.

## 5 Extrafollicular B cell responses in autoimmunity

In recent years, it has been increasingly recognized that the extrafollicular B cell response is an important source of pathogenic autoantibodies, and that, both in autoimmune-prone mouse models (see Table 1) and in patients with autoimmune diseases (Jenks et al., 2018;Ota et al., 2023), extrafollicular autoantibodies are a key causative factor in the pathogenesis of autoimmune diseases.

### 5.1 ABCs in autoimmunity

ABCs constitute an increasingly recognized extrafollicular subpopulation (Jenks et al., 2018;Song et al., 2022), initially named after observing their accumulation with age in humans and mice (Hao et al., 2011). Their accumulation is also evident in autoimmune diseases. ABCs are also referred to as atypical memory B cells and double negative 2 (DN2) in humans, due to lack of IgD and CD27 expression. ABCs are characterized by high expression of CD11c and/or CD11b, FcRL5, and CD19 and low expression of IgD, CD27, CD21, and CXCR5. Their differentiation is driven by the transcription factors T-bet and ZEB2 (Dai et al., 2024;Gao et al., 2022;Jenks et al., 2018;Nickerson et al., 2023;Song et al., 2022). ABC generation requires T cell help (Odegard et al., 2008;Song et al., 2022) and is mediated by signals through the B cell receptor (BCR), TLR7 expressed in B cells, and cytokines such as IFN-γ and IL-21 (Brown et al., 2022;Jenks et al., 2018;Nickerson et al., 2023;Rubtsov et al., 2011;Wang et al., 2018) (Figure 1).

ABCs have been implicated in autoimmune diseases including systemic lupus erythematosus (SLE) (Jenks et al., 2018;Nickerson et al., 2023;Rubtsov et al., 2011), rheumatoid arthritis (RA) (Qin et al., 2022) and Sjögren’s Syndrome (Saadoun et al., 2013). The frequency of circulating ABCs in human SLE correlates with autoantibody titres (e.g., anti-nuclear, anti-dsDNA, anti-RNA, and anti-chromatin), and disease severity measured by the SLE Disease Activity Index (SLEDAI) (Jenks et al., 2018;Wang et al., 2018;Wu et al., 2019). ABCs are particularly expanded in patients with lupus nephritis (LN), and negatively correlate with renal impairment (He et al., 2022;Sosa-Hernandez et al., 2022). It has been established that ABCs readily differentiate into PCs, and their absence results in reduced autoantibodies and histologic manifestation (Dai et al., 2024;Nickerson et al., 2023;Rubtsov et al., 2011). ABCs appear to contribute to both systemic inflammation and organ damage in autoimmunity. The precise mechanisms underlying ABCs’ function and their intricate interactions with other immune cells including conventional extrafollicular plasmablasts are still being elucidated, holding much promise for the development of targeted therapies for autoimmune diseases (Levack et al., 2022).

### 5.2 Extrafollicular T cell help

Depending on the absence or presence of T-cell help, extrafollicular B cell responses can be categorized into either the thymus-independent (TI) (Garcia de Vinuesa et al., 1999;Hsu et al., 2006;Martin, Oliver and Kearney, 2001) or thymus dependent (TD) pathways (Jang et al., 2019;Keller et al., 2021;Odegard et al., 2008;Rao et al., 2017), with the latter being prominent in autoimmune conditions.

#### Extrafollicular T helper (EF T_H_) cells

It is now recognized that Bcl6+ T_FH_ cells first localize at the T-B border, where they prime B cells prior to their commitment to the GC or extrafollicular route of differentiation (Lee et al., 2011). Such extrafollicular or pre-GC T_FH_ cells not only provide help for B cell to differentiate into to extrafollicular PCs (Kim et al., 2018;Lee et al., 2011), but are also necessary for the generation of pathogenic ABCs (Song et al., 2022). In addition, there is a group of ICOS+ T_FH_-like cells detected at extrafollicular sites that promote antibody responses via IL-21 and CD40L (Odegard et al., 2008;Poholek et al., 2010). Therefore, overactive T_FH_ cells or T_FH_-like cells can also cause autoimmunity through an extrafollicular pathway.

#### Peripheral T helper (T_PH_) cells

In non-lymphoid tissues (e.g., blood and synovium), a subset of PD1^hi^ CXCR5^-^ CD4 T cells, known as T_PH_ cells, are largely expanded in patients with autoimmune diseases such as RA (Rao et al., 2017) and SLE (Caielli et al., 2019;Ota et al., 2023). These cells guide the differentiation of extrafollicular B cells through IL-21 (Rao et al., 2017) or IL-10 (Caielli et al., 2019). To date, mechanisms underlying T_PH_ development and pathogenesis have not been fully elucidated, and the relationship between T_PH_ and conventional T_FH_ cells remains largely unknown. Nevertheless, targeting extrafollicular T_PH_ responses is being considered for treatment of autoimmune diseases.

## 6 Concluding remarks

It has been controversial for many years whether GCs in autoimmunity are pathogenic or protective. Evidence has continued to emerge in recent years suggesting that GCs are likely to be dispensable for the initiation of systemic autoimmune diseases, and in contrast, the extrafollicular pathway is sufficient to drive autoimmunity. Despite the risk of SHM in GCs generating self-reactive B cells, in healthy individuals these cells can remain confined to GCs and are either cleared by apoptosis, mutate away from autoreactivity, or are prevented from differentiation by the absence of self-reactive T_FH_ cells and presence of T_FR_ cells. Thus, at least for systemic autoimmunity, GC seems to be the gatekeeper of self-tolerance, rather than the kicker. An in-depth understanding of how to attain removal of self-reactivity in GCs while preventing Tlr7-driven self-reactive extrafollicular responses will be valuable in the fight against autoimmune diseases.

## Figures and Tables

**Table 1:** Roles of GCs in mouse models of autoimmunity.

Mouse strain	Manifestations	GC formation	Autoantibody origin	Driven by TLR7?	Reference
Systemic autoimmunity without formation of spt-GCs					
cGVHD	Scleroderma, lymphocytic bronchitis, damage in the salivary and lacrimal glands, Anti-dsDNA-IgG and IgG deposition in the skin and thymus.	No or less GCs	T cell-dependent extrafollicular pathway	Not tested	(Deng et al., 2017)
C57BL/6 →BALB/c					
B cell-condition al *Ikzf1* deficiency (*Ikzf1*^B-^)	Splenomegaly, systemic inflammation, ANA.	No GCs	Myd88-dependent extrafollicular pathway	Not tested	(Schwickert et al., 2019)
*Bach2-/-*	Lupus nephritis, elevated titers of IgG-switched autoantibodies	Defects in GC formation	T cell-dependent extrafollicular pathway	Not tested	(Jang et al., 2019)
B cell-intrinsic Ship-deficiency (*Ship*^B-^)	Excessive autoantibodies against ssDNA, dsDNA, dsRNA and chromatin.	Impaired GC B responses	Extrafollicular ABCs contributes to autoantibody production	Not tested	(O’Neill et al., 2011;Zhang et al., 2019)
*Dnase1l3-/-*	Glomerulonephritis, splenomegaly, rapid anti-dsDNA antibody responses.	Normal	IFN-I producing pDCs facilitate extrafollicular autoantibody responses.	TLR7 and TLR9	(Soni et al., 2020)
Autoimmunity with spt-GCs					
*TLR7^Y264H^*,	Thrombocytopenia, proliferative glomerulonephritis, lymphoid infiltrates, splenomegaly, decreased survival and ANAs with nuclear.	Increased GCs	GC B cells are dispensable for the autoimmune phenotype and instead protect against it, suggesting that the pathogenic autoantibody response originates from the EF pathway.	Yes	(Brown et al., 2022)
*TLR7^Y264H^* *Bcl6^flox^ and* *TLR7^Y264H^ Bcl6^flox^ Cd23^cre^*					
*564Igi* and	Mesangioproliferative glomerulonephropathies, IgG autoantibodies	Increased GCs	GC block fail to mitigate autoimmunity, suggesting an extrafollicular origin of autoantibody responses.	Yes	(Berland et al., 2006;Voss et al., 2022)
*564Igi* *Aicda^Cre^* *Bcl6^flox^*					
R848 induced lupus	Splenomegaly, dsDNA IgG2c, immune complex deposition in glomeruli.	Increased GCs	EF response is sufficient to drive autoimmunity.	Yes	(Voss et al., 2022)
*Aicda^Cre^* and *Aicda*^Cre^ *Bcl^flox^*					
MRL.Fas lpr (MRL/lpr)	Lymphoproliferation, progressive renal failure, Lymphadenopathy and skin lesions, autoantibodies including ANA, anti-dsDNA, anti-Sm, anti-Ro and anti-La.	Increased in young mice but not in old mice	Autoantibodies are generated by somatic hypermutation at the T zone–red pulp border rather than in GCs.	Yes	(Cosgrove et al., 2023;William et al., 2002)
NZM2410-derived B6.Sle1.Sle2.Sle3 (TC) strain	Systemic autoimmunity with fatal glomeruloneph ritis, increased anti-chromatin and anti-dsDNA IgG2a.	Increased GCs	The EF foci rather than the GC are positively correlated with the production of autoantibodies	Partially	(Sang et al., 2014)
BAFF tg	Systemic autoimmunity with nephritis and salivary gland destruction	Numerous GCs	Shown to be T-independent, and thus likely extrafollicular	Driven by MyD88 and TLR7/9	(Groom et al., 2007)

Abbreviations: ANA, antinuclear antibodies; dsDNA, double-stranded DNA; Sm, Smith; ssDNA, single-stranded DNA; GC, germinal center; GVHD, Graft-versus-host disease; ABC, age-associated B cell; pDCs, plasmacytoid dendritic cells; IFN-I, Type I interferon.

**Table 2:** B cell self-tolerance checkpoints in GC

Factors	Mechanisms	Reference
B cell negative selection in germinal centers	BCR-mediated clonal deletion.	(Pulendran et al., 1995;Smith et al., 2000)
	FAS-mediated cell apoptosis.	(Butt et al., 2015;Smith, Nossal and Tarlinton, 1995)
	T_FR_-mediated inhibition of self-reactive B cell differentiation into plasma cells.	(Chung et al., 2011;Jacobsen et al., 2021;Linterman et al., 2011;Wollenberg et al., 2011)
	Macrophage clearance of apoptotic cells.	(Grootveld et al., 2023;Hanayama et al., 2004).
Control of positive GC B cell selection	Limiting T_FH_ cell help for B cells presenting self-peptide.	(Merkenschlager et al., 2021;Vinuesa et al., 2005)
	Self-reactive GC B cells are not selected into the memory pool.	(Cappione et al., 2005;Richardson et al., 2013)
B cell confinement in germinal centers	GPCRs (e.g., S1PR2 and P2RY8) promote B cells to be confined within GC.	(He et al., 2022;Lu et al., 2019;Muppidi, Lu and Cyster, 2015)
Autoreactivity redemption by SHM	Self-reactive B cells mutate away from self-reactivity in GCs.	(Burnett et al., 2018;Sabouri et al., 2014)

Abbreviations: BCR, B cell receptor; GC, germinal center; GPCR, G protein-coupled receptors; SHM, somatic hypermutation.
